# Development and preliminary evaluation of a German version of the active communication education group aural rehabilitation training program

**DOI:** 10.3389/fresc.2026.1828976

**Published:** 2026-07-10

**Authors:** Sybille Seybold, Frauke Koppelin, Nerina Scarinci, Louise Hickson

**Affiliations:** 1 Jade University of Applied Sciences, Department of Construction Geoinformation Health Technology, Section Technology and Health for Humans, Oldenburg, Germany; 2 Centre for Hearing Research (CHEAR), School of Health and Rehabilitation Sciences, The University of Queensland, Brisbane, DE, Australia

**Keywords:** acceptance, active communication education, age-related hearing loss, communication strategies, group aural rehabilitation program

## Abstract

**Objective:**

This study investigated the effectiveness of a German version of the Active Communication Education (ACE) program.

**Research design:**

An exploratory cohort study was conducted with pre-post measures of communication strategy use, acceptance, activity and participation, health-related quality of life, anxiety, and depression. Post-program measures also included the International Outcome Inventory – Alternative Interventions (IOI-AI), a modified version of the Client Oriented Scale of Improvement (M-COSI), and open-ended questions.

**Study sample:**

Thirty older adults with acquired hearing loss (*M* age = 70.27, *SD* = 5.77) attended three or more ACE sessions.

**Intervention:**

The ACE program consisted of five, weekly 2 h group sessions with 6 to 10 participants.

**Data collection and analysis:**

Pre-post self-report measures were completed pre-program, after two weeks, and 6 months post-program. Within-group changes and effect sizes were calculated. The IOI-AI results were descriptively analysed. Categories for the M-COSI and open-ended data were analysed using the qualitative method of summarizing-content analysis.

**Results:**

Statistically significant within-group improvements were found for communication strategy use and acceptance of hearing loss. The IOI-AI showed a positive impact of ACE on activity and participation. The majority of participants (83.3%) reported improvement in their primary communication goal.

**Conclusion:**

The German version of the ACE program showed positive benefits for adults with hearing loss and can be recommended as a rehabilitative intervention for the target population.

## Introduction

Through the effective use of communication strategies, everyday communication can be improved for adults with hearing loss (AHL). For those who do not or cannot wear hearing aids, communication training programs are an effective alternative to hearing aid fitting, and they can supplement hearing aids for people who continue to experience communication difficulties in everyday life even with hearing aids. Thus, to support AHL a number of communication training programs have been developed and evaluated (1–5). Typically, such programs have been found to reduce hearing-related communication difficulties (2, 5–8).

The focus of the current research described here, was the Active Communication Education (ACE) program developed by Hickson, Worrall and Scarinci (9), Hickson, Worrall and Scarinci (10). When initially developed in Australia it was evaluated in a randomized, controlled trial with 178 study participants (96 hearing aid users, 82 non-users). Hickson, Worrall, and Scarinci (2) compared ACE to a placebo social intervention, and compared to the control group, those who participated in ACE reported significant reductions in activity limitations and participation restrictions, and an improvement in well-being. Subsequently, the ACE was translated into other languages, with evaluations reported from Sweden and Chile. Two studies related to the Swedish ACE version found significant improvements for older adults with hearing loss in terms of communication strategy use and a reduction of perceived hearing disability (5, 7). Likewise, for the Spanish version evaluated in Chile, Rivera et al. (8) reported significant improvements in hearing function, and less hearing disability in daily life for adults who attended ACE. This indicates that translations of ACE have value in terms of providing an intervention for AHL from different cultural and linguistic backgrounds.

In Germany, there is a lack of evidence-based communication training programs for AHL. In a representative German study, von Gablenz and Holube (11) found that 20% of people aged 60-69 years had hearing loss, with this prevalence increasing to 41.3% for people aged 70−79 years. After the diagnosis of hearing loss, hearing aid fitting, if indicated, is often the only healthcare service offered to adult outpatients (12). Therefore, there is a clear need for additional support services, and, given the evidence supporting the effectiveness of the ACE program in other languages, permission was sought to develop a German version.

The overall objective of the study was to develop a version of ACE which met the needs of older AHL in Germany. The study also aimed to investigate outcomes of this German version of ACE for AHL by evaluating self-reported (1) pre-post changes in the use of communication strategies, activity and participation, acceptance of hearing loss, health-related quality of life, and anxiety and depression, and (2) post-program outcomes.

## Methods

### Participants

Participants were recruited from the database of Hörzentrum Oldenburg gGmbH (collaborating hearing research institute) from public presentations about hearing-related communication difficulties, and through distribution of flyers to hearing-aid dispensers and otolaryngologists. Individuals on the database were contacted by phone, and potential participants who heard about the study via flyer or presentation contacted Jade University via phone or email.

**Figure 1:**
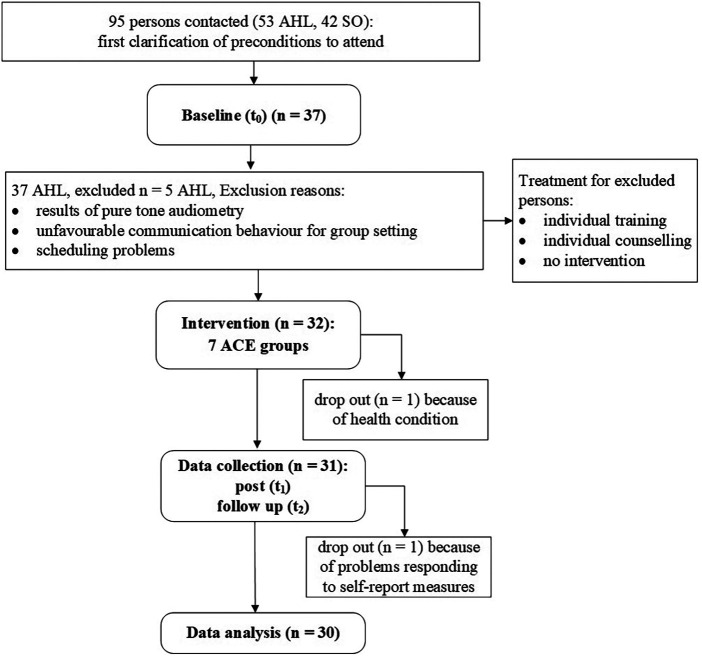
Summary of study participant involvement (AHL = persons with hearing loss, SO = significant others) with measurement points t_0_ (baseline), t_1_ (post - two weeks after the last ACE session), t_2_ (follow-up - six months after the last ACE session).

A sample size of at least 20 participants was targeted, based on an effect size of 0.6 for the Communication Strategies Scale (CSS) of the Communication Profile for the Hearing Impaired (CPHI) of Kricos and Holmes (13) within audiological rehabilitation. A total of 53 potential participants were subsequently contacted by phone, at which time they were given information about the ACE program, data-collection procedures, and scheduling aspects. Thirty-seven AHL expressed an interest in participating in the study and were invited to Jade University for an introductory appointment that lasted approximately 2 h (see Figure 1). It included pure-tone audiometry based on the DIN ISO 8253-1 (14) standard in a process with ascending level, more detailed information about the ACE program, informed consent (see paragraph procedure) and guideline-based anamnesis related to inclusion and exclusion criteria.

Inclusion criteria for the AHL were: (1) aged 60 to 80 years, (2) acquired hearing loss with at least 26 dB HL PTA-4 (0.5, 1, 2 and 4 kHz of the better ear), (3) self-reported hearing-related communication difficulties, and (4) self-reported motivation to work on hearing-related communication difficulties. Exclusion criteria were: (1) not being able to function effectively in a group setting as a result of severe communication difficulties (self-reported by AHL or observed by the first author) and (2) self-reported cognitive problems.

If interested participants did not fulfil the study inclusion criteria, but it was indicated that the ACE program would be helpful, individualized ACE sessions were provided for ethical reasons.

The final study sample comprised 30 AHL (see Table 1). All participants had German as their first language and had good written language skills. Nineteen significant others attended the program and all were spouses of AHL. AHL who attended without another person reported that they lived alone. Seven groups were formed for participation in the ACE program (based on participant availability). Most AHL participated in all 5 sessions (20 AHL), while 9 participated in 4 sessions and one AHL participated in only 3 sessions.

**Table 1:** Demographic data and hearing difficulties of study participants.

*Variable*	Frequency/Percentage	
*Age*	Mean	70.27
	SD	5.77
	Range	59–79
*Gender*	Women (%)	22 (73%)
	Men (%)	8 (27%)
*Hearing loss*		
PTA-4	Mean	45.74 dB HL
	SD	19.25 dB HL
	Range	26.25–120 dB HL
Mild HL	Number (%)	14 (47%)
Moderate HL	Number (%)	11 (37%)
Severe HL	Number (%)	4 (13%)
Profound HL	Number (%)	1 (3%)
*Hearing devices*		
Nonuser	Number (%)	4 (13%)
Hearing-aid user	Number (%)	25 (83%)
Binaural hearing-aid user	Number (%)	22 (73%)
Cochlear-implant user	Number (%)	1 (3%)
Binaural cochlear-implant user	Number (%)	1 (3%)
*Daily hearing-aid use*		
1–4 h/day	Number (%)	2 (7%)
5–8 h/day	Number (%)	5 (17%)
>8 h/day	Number (%)	18 (60%)

HL, Hearing loss; PTA-4, Pure-Tone Average of the better ear for the frequencies 0.5,1,2,4 kHz., WHO (15) criteria for grades of HL based on PTA-4 of the better ear: no HL: 25 dB HL or better, mild HL: 26–40 dB HL, moderate HL: 41–60 dB HL, severe HL: 61–80 dB HL, profound HL: 81 dB HL and more. Duration of daily hearing aid use was self-reported.

### Outcome measures

The following self-report questionnaires were chosen for this study based on their use in previous ACE evaluations and their availability as validated German versions.

#### Pre-post program measures

The following measures were used at pre-program (t_0_), post-program (t_1_), and 6 months follow-up (t_2_).

**The Communication Strategies Scale (CSS) and the Acceptance Scale** are part of the Communication Profile for the Hearing Impaired (CPHI (16);. The CSS explores the use of communication strategies and was previously used to evaluate the Swedish ACE (5–7). Wisotzki and Mühlich (17) translated the CPHI into German, adapted it to German socio-cultural aspects, and subsequently validated it. The German CSS has 4 items fewer than the English and Swedish versions. Five items were deleted for statistical reasons, and based on interviews conducted during the development and validation of the German CPHI it was found important to add the item: “I ask others to speak more slowly”. The German CSS includes three subscales, with 21 items in total: 7 verbal items (CSS V), 8 non-verbal items (CSS NV), and 6 maladaptive items (CSS M).

The Acceptance Scale of the CPHI evaluates the acceptance of personal hearing loss.

It includes two subscales with 11 items in total: Self-Acceptance Subscale (Self-Accept) which includes feelings and attitudes of the AHL towards their own hearing loss (5 items), and the Acceptance Subscale (Accept) that relates to self-disclosure of one’s hearing loss to others (6 items).

In contrast to the original version of this measure which had a 5-point response scale, the German version has a 4-point response scale. Response options were never (1), rarely (2), sometimes (3), and always (4) *or* strongly agree (1), agree (2), disagree (3), and strongly disagree (4). Higher scores indicate a better/more effective use of communication strategies and more positive acceptance. Cronbach's *α* for the subscales of the German version range from.73 to.84, and indicate acceptable or good internal consistency (17).

**The Hearing Handicap Inventory for the Elderly/Adults (HHIE/A)** consists of 25 items divided into two subscales: (1) emotional hearing handicap (13 items), and (2) social hearing handicap (12 items). The HHIE/A provides information about the activity and participation of AHL (18–20). The item number and subscales are the same for the HHIE and HHIA, but three items were substituted to better address hearing needs of adults who remain in the work-force (19). The two different versions of the questionnaire (HHIE and HHIA) were joined, and as in other studies, data calculated for both versions together (21, 22). The response scale has three options: no (0), sometimes (2), and yes (4), with higher scores indicative of greater perceived hearing difficulties. The item number and subscales are the same for the HHIE and HHIA. In the HHIA, however, three items are substituted to better address the hearing needs of AHL who remain in the workforce (19, 23). The German versions of the HHIE/A were developed and validated by Tesch-Römer (24) and have good-to-high internal consistency reliability (2001, S.66ff). The HHIE was previously used to evaluate the Swedish (5–7) and Spanish ACE versions (8).

**The Short-Form 12 (SF-12)** has 12 items measuring health-related quality of life, and is a shortened version of the SF-36. The German versions of the SF-36 and SF-12 were developed by Morfeld et al. (25). The same 8 domains as in the SF-36 are covered by the SF-12: physical functioning, role-physical, bodily pain, general health, vitality, mental health, role-emotional, and social functioning. Results can be calculated for a mental component score and a physical component score, and values range from 0 to 100; higher scores indicate better health-related quality of life. Cronbach's *α* values for the German version were assessed as acceptable or good, with a range from.70 to.87. The SF-36 was used to evaluate the original ACE by Hickson et al. (2).

**The Hospital Anxiety and Depression Scale (HADS)** (26) is a 14-item measure designed as a brief screen to assess depression (7 items) and anxiety (7 items). There is a 4-point response scale, with values from 0 to 3 (total score range = 0 to 21). Higher scores indicate more anxiety and/or depression symptoms. The German Version (HADS-D) was developed by Herrmann-Lingen, Buss, and Snaith (27) and has good retest-reliability (>.8) and good internal consistency, with cronbach's *α* = .80 (anxiety) and.81 (depression). The HADS was used in previous evaluation studies of the Swedish ACE (5, 6).

#### Post-Program measures

The following measures were used at post-program (t_1_), and at the 6-month follow-up (t_2_).

**The International Outcome Inventory for Alternative Interventions (IOI-AI)** consists of seven items and a 5-point rating scale. Higher scores indicate a better outcome. Each question refers to a specific outcome: 1. daily use of ACE strategies, 2. benefit, 3. residual activity limitations, 4. satisfaction with the program, 5. residual participation restrictions, 6. impact on others, and 7. impact on the quality of life (28) and has previously been used for the ACE program (5, 6, 29). Good internal consistency was found by Hickson, Worrall and Scarinci (29). A German version of the IOI-AI was derived from the available German version of the International Outcome Inventory – Hearing Aids (IOI-HA) (30). The IOI-AI was suggested as an outcome measure after the ACE program in the ACE handbook (9, 10) and was used in previous ACE intervention studies (2, 5).
**In the first session, a modified version of the Client Oriented Scale of Improvement (M-COSI)** (2) was used to identify hearing or communication goals of study participants. Every person wrote down their own goals in a two- step-approach. In the first step, participants were asked to write down up to four hearing-related communication difficulties, being as specific as possible. In the second step, participants were asked to prioritise which communication difficulties they wanted to improve most. The prioritised difficulties were considered as individual communication goals and registered in the M-COSI response sheet by the course facilitator. At t_1_ and t_2,_ participants then chose one of the following responses in relation to each goal: worse (1), no difference (2), slightly better (3), better (4), and much better (5). The M-COSI only contains the scale “degree of change”, in contrast to the original COSI, which has a second scale “final ability (with hearing aid)” (31). The items were generated by AHL themselves, therefore only translation (by the first author) into German was necessary for the response scales. The M-COSI was also suggested as an outcome measure after the ACE program in the ACE handbook (9, 10) and was used in previous ACE intervention studies (2, 5).
**Feedback Sheets:** Feedback sheets were completed at the end of each session and after the whole program. The questions were “What did you like about the ACE session/s?” and “How could the ACE session/s be improved?”

### Procedure

The study and protocol were reviewed and approved by the Research Ethics Committee of the Carl von Ossietzky University, Oldenburg. Written informed consent was obtained from all participants in accordance with the Declaration of Helsinki. Potential participants interested in joining the whole program were invited to an appointment at Jade University. After informed consent was obtained, demographic information and baseline data were collected via questionnaires.

To determine eligibility, pure-tone audiometry based on the DIN ISO 8253-1 (14) standard in a process with ascending level was conducted by hearing professionals. The facilitator of all ACE sessions was the first author, who has a professional background as a speech-language therapist and health scientist. The sessions were supported by an assistant with a professional background as an audiologist. The ACE sessions took place at the Jade University in Oldenburg. The study participants received no financial compensation for their participation. They were, however, offered participation in a communication training program including all materials and catering during the break within each ACE session. All participants were personally invited to the Jade University for all three measurement points completed pre-program (t_0_), after two weeks post-program (t_1_), and after 6 months follow-up (t_2_). AHL completed all questionnaires themselves, but were also encouraged to ask questions of the researchers if they needed assistance. An independent professional attended participants while they completed the questionnaires.

Outcomes for significant others were also collected during this research, but these are not reported here.

### ACE intervention

All materials of the original ACE program (9) were carefully translated into German in a step-by-step approach: (1) translation of ACE materials from English into German, (2) first review, (3) first corrected version, (4) research stay at the University of Queensland where the first author participated in an ACE tutorial for students, attended ACE sessions, and discussed translational aspects of the program with ACE authors (5) second corrected version, (6) second review and (7) third corrected version (used in the final study). Reviewers were bilingual English-German and had bicultural Australian-German backgrounds.

The ACE program consists of five weekly 2-hour sessions, each including a break of 15 min. The first session starts with an analysis of communication needs and the identification of individual hearing and communication goals. The following four sessions are then selected based on the analysis of communication needs analysis and each session focuses on one relevant goal. Topics of the sessions typically include: conversation in background noise, communication with difficult speakers, conversations around the house, listening to other signals and public address-systems, and speech reading. The aim of the ACE program is for participants to learn and internalize problem-solving skills that make it possible for them to manage a range of challenging communication situations by modifying their own behavior. Significant others of AHL were also included, as managing communication issues frequently requires support from communication partners. A copy of German ACE (ZAK – Zusammen Aktiv Kommunizieren) is available on request from the first author. More information about the ACE program is available under https://shrs.uq.edu.au/active-communication-education-ace.

### Data analysis

The statistical software package SPSS (IBM SPSS Statistics Version 20) was used for analysis. For the pre-post program measures, within-group comparisons were conducted using parametric statistics (ANOVA) when the Shapiro–Wilk-Test indicated a normal distribution; nonparametric statistics (Friedman ANOVA) were used when this precondition was not met. *post hoc* tests were conducted to identify statistically significant changes at different time points. Descriptive data, within-group changes over time, and the effect size Cohen's *d* were calculated for all pre-post program measures by dividing the difference between pre- and post- mean scores by the pre-mean standard deviation (32). The effect size Cohen's *d* is interpreted with values of 0.2 or higher as a small, and 0.5 or higher as a moderate effect. Post-program measures were analysed descriptively. Qualitative data of individual goals on the M-COSI and the feedback sheets were analysed by using the summarizing-content analysis of Mayring (33) and the software Maxqda (Maxqda 11). Summarizing-content analysis is a rule-guided text analysis, which aims at a categorical system to summarize and reflect the original text material. In a first step, the text material was worked through carefully: Context units were identified and condensed using a defined procedure of paraphrasing, generalizing, and labelling with codes that lead to inductive categories. The final category system was based on an inductive, circular process. All categories were defined, by means of prototypical text passages, and described by coding rules for distinguishing between categories. A coding conference was conducted with two scientists of adjacent professions (person 1: health sociologist, person 2: health and nursing scientist) who were experienced in qualitative data analysis.

## Results

### Results for pre-post program measures

Descriptive results for all pre-post measures are shown in Table 2. The results of the statistical analysis of within-group effects are summarized in Table 3.

**Table 2:** Descriptive data for pre-post program measures at pre (t_0_), post (t_1_) and follow-up (t_2_) (*N* = 30).

	t_0_			t_1_			t_2_		
Questionnaire	*M*	*SD*	*Range*	*M*	*SD*	*Range*	*M*	*SD*	*Range*
CPHI									
CSS V	19.20	3.07	12–25	20.47	3.50	13–26	19.80	3.20	14–28
CSS N	23.30	4.24	13–31	24.53	3.95	16–31	24.80	4.07	16 −32
CSS M	11.13	3.20	13–33	10.47	2.65	6–16	10.83	3.10	6–19
CSS total	61.37	6.39	13–34	64.53	7.56	48–76	63.77	7.36	53–82
Self-Accept	14.43	3.13	13–35	15.53	2.89	7–20	15.67	2.96	8–20
Accept	19.50	3.90	13–36	20.40	3.01	14–24	20.53	3.36	12–24
Accept total	33.93	6.44	13–37	35.93	5.37	22–43	36.20	5.76	20–43
HHIE/A									
HHIE/A E	19.80	11.83	0–48	18.87	11.41	0–46	16.73	10.71	0–38
HHIE/A S	18.53	11.60	2–40	17.50	11.15	4–42	16.13	9.71	2–34
HHIE/A total	38.33	22.78	4–88	36.37	21.63	4–88	32.87	19.79	4–70
SF-12									
SF-12 P	49.13	8.26	32.84–62.41	47.25	10.86	27.28–66.04	44.86	11.84	20.78–64.21
SF-12 M	46.73	10.58	22.03–61.55	43.45	12.89	19.98–59.16	45.48	11.62	22.99–61.78
SF-12 total	95.86	12.66	54.87–109.62	90.69	16.75	51.20–115.30	90.33	16.38	52.06–112.68
HADS-D									
HADS-D D	4.27	3.08	1–12	4.97	3.52	1–14	4.97	2.91	1–11
HADS-D A	5.63	3.31	1–14	7.17	4.22	0–15	6.53	4.01	0–16
HADS-D total	9.90	5.94	3–26	12.13	7.44	1–29	11.50	6.54	1–27

Descriptive data with means (*M*), standard deviation (*SD*) and range for all pre-post measurs. CSS, Communication Strategies Scale of the Communication Profile for the Hearing Impaired (CPHI); CSS V, Verbal Communication Strategies Subscale of the CSS; CSS N, Nonverbal Communication Strategies Subscale of the CSS; CSS M, Maladaptive Communication Strategies Subscale; Self-Accept, Self-Acceptance Subscale of the CPHI; Accept, Acceptance towards others Subscale of the CPHI; HHIE/A, Hearing Handicap Inventory for the Elderly/Adults; HHIE/A E, Emotional Subscale of the HHIE/A; HHIE/A S, Social Subscale of the HHIE/A; SF-12, Short Form 12; SF-12 P, Physical Component Scale of the SF-12; SF-12 M, Mental Component Scale of the SF-12; HADS-D, German Version of the Hospital Anxiety and Depression Scale; HADS-D D, Depression Scale of the HADS-D; HADS-D A, Anxiety Scale of the HADS-D.

**Table 3:** With-in group effect with F- or *χ*^2^- values (*N* = 30). Significant differences are marked in bold.

Questionnaire	F-value/*χ*2- values 1–3	Post hoc t_0_-t_1_	Post hoc t_0_ t_2_	Post hoc t_1_-t_2_
CPHI				
CSS V	*F*(2,58) = 2.58, ns (*p* = .084)	*-*	*-*	*-*
CSS N	*F*(2,58) = 2.75, ns (*p* = .072)	*-*	*-*	*-*
CSS M	*F*(2,58) = 1.58, ns (*p* = .214)	*-*	*-*	*-*
CSS total	*F*(2,58) = 5.43, ***p*** **=** **.007**	***p*** **=** **.001**	***p*** **=** **.043**	*p* = .430
Self-Accept	χ^2^ (2) = 6.88, ***p*** **=** **.032**	***p*** **=** **.028**	*p* = .061	*p* = .747
Accept	χ^2^ (2) = 3.31, ns (*p* = .192)	*-*	-	-
Accept total	χ^2^ (2) = 6.07, ***p*** **=** **.048**	***p*** **=** **.039**	***p*** **=** **.045**	*p* = .949
HHIE/A				
HHIE/A E	*F*(2,58) = 2.40, ns (*p* = .100)	*-*	*-*	*-*
HHIE/A S	*F*(2,58) = 1.73, ns (*p* = .189)	*-*	*-*	*-*
HHIE/A total	*F(2,58)* *=* 2.54, ns *(p* *=* .088)	*-*	*-*	*-*
SF-12				
SF-12 P	χ^2^ (2) = 2.67, ns (*p* = .264)	*-*	*-*	*-*
SF-12 M	χ^2^ (2) = 1.84, ns (*p* = .397)	*-*	*-*	*-*
SF-12 total	χ^2^ (2) = 2.94, ns (*p* = .230)	*-*	*-*	*-*
HADS-D				
HADS-D D	χ^2^ (2) = 2.53, ns *(p* = .282)	-	-	-
HADS-D A	χ^2^ (2) = 6.13, ***p*** **=** **.047**	***p*** **=** **.024**	*p* = .107	*p* = .519
HADS-D total	χ^2^ (2) = 5.02, ns (*p* = .081)	-	-	-

F-values (ANOVA for parametric data analysis) and χ2- values (Friedman ANOVA for non-parametric data analsis, with asymptotic significance) of within-subject-effect for factor time. Two-sided significance level ˂.05. ns, no statistical significance. Post hoc-tests: LSD (least significant difference).

### The communication strategies scale (CSS) and the acceptance scale

#### Communication strategy use

Statistically significant improvements were found for CSS total scores [*F* (2,58) = 5.43, *p* = .007], indicating that after attending the program, participants used more communication strategies to enhance their everyday communication. *post-hoc* testing indicated that differences were statistically significant between t_0_ and t_1_ (*p* = .001), and between t_0_ and t_2_ (*p* = .043). The effect size Cohen's *d* was moderate between t_0_ and t_1_ [*d* = 0.50, 95% CI (−0.23–1.22)], and small between t_0_ and t_2_ [*d* = 0.38, 95% CI (−0.35–1.10)]. The effect sizes showed positive effects of more frequently used communication strategies.

#### Acceptance of hearing loss

For the Accept total score, a statistically significant within-group effect was found [*χ*^2^(2) = 6.07, *p* = .048]. This indicates that participants were better able to accept their hearing loss after completing the ACE, and reported more positive feelings and attitudes towards their hearing loss, and increased readiness to disclose their hearing loss or hearing-related difficulties. *post-hoc* tests showed that statistically significant differences occurred from t_0_ to t_1_ (*p* = .039,) and from t_0_ to t_2_ (*p* = .045). Cohen's *d* indicated small effect sizes between t_0_ and t_1_ [*d* = 0.31, 95% CI (−0.41–1.03)], and t_0_ and t_2_ [*d* = 0.35, 95% CI (−0.37–1.07)]. The effect sizes indicated only small effects of the enhanced acceptance of hearing loss.

### Hearing related social and emotional difficulties

25 AHL were retired and completed the HHIE, five participants were working part-time or full-time and completed the HHIA. No statistically significant differences were found for the total HHIE/A scores and for both social and emotional subscales across the three time points.

### Health-related quality of life, anxiety and depression

No statistically significant differences were evident over time using the SF-12, indicating no change in self-reported health-related quality of life following participation in the German ACE program. The Anxiety subscale of the HADS-D showed a statistically significant within-group effect of time [*χ*^2^ (2) = 6.13, *p* = .047], indicating that anxiety increased over time. *post-hoc* testing showed that values on the anxiety subscale increased most from t_0_ to t_1_ (*p* = .024) and a small effect with a Cohen's *d* value of = 0.47 [95% CI (−0.26–1.91)] was found. This reflects an effect of increased anxiety between t_0_ and t_1._ The effect decreased over time from t_0_ to t_2_ [*d* = 0.27, 95% CI (−0.45–0.99)].

#### Results for post-program measures

##### IOI-AI results

The IOI-AI results indicated positive experiences post program, particularly in terms of benefit (item 2), residual activity limitations (item 3), satisfaction (item 4), residual participation restriction (item 3 + 5), and impact on others (item 6) (see Table 4). The majority of participants reported that they used the ACE strategies ‘1-4 h per day’ (item 1). The results at t_1_ and t_2_ showed no statistically significant difference for any item or for the total scores (asymptotic Wilcoxon-Test: *z* = −1.44, *p* = .15).

**Table 4:** Results for each item of the IOI-AI at t_1_. Percentage distribution and descriptive data (*N* = 30).

Item 1: Use		Item 2: Ben		Item 3: RAL		Item 4: Sat		Item 5: RPR		Item 6: Ioth		Item 7: QoL	
None	-	Not at all	-	Very much	-	Not at all	-	Very much	-	Very much	-	Worse	-
<1 h/day	36.70%	Slightly	20.00%	Quite a lot	13.30%	Slightly	-	Quite a lot	3.30%	Quite a lot	-	No change	6.70%
**1–4 h/day**	**56.70%**	Moderately	16.70%	**Moderate**	**73**.**30%**	Moderately	3.30%	Moderate	20.00%	Moderately	3.30%	**Slightly**	**53**.**30%**
4–8 h/day	6.70%	**Quite a lot**	**53.30%**	Slight	13.30%	Quite a lot	43.30%	**Slight**	**46.70%**	Slightly	36.70%	Quite a lot	33.30%
>8hr/day	-	Very much	10.00%	None	-	**Very much**	**53**.**30%**	None	30.00%	**Not at all**	**60**.**00%**	Very much	6.70%
*M*	2.70		3.53		3.00		4.50		4.03		4.57		3.40
*SD*	0.60		0.94		0.53		0.57		0.81		0.57		0.72

Ben, benefit; RAL, residual acitivity limitations; Sat, satisfaction; RPR, residual participation restriction; Ioth, impact on others; QOL, quality of life. Most frequent answer in **bold**. Scores range from 1 up to 5. Higher scores stand for better outcome.

#### Individual hearing and communication goals

Participants generated a total of 118 hearing and communication goals. Nine goals were excluded as irrelevant, leaving 109 comments. Excluded goals were determined to be either too general or not related to the ACE program [e.g., “*noisy surrounding (also movies/TV, traffic)”*]. Seven main goals were evident (see Table 5). The most common goal related to ‘*adverse speech and communication behavior of communication partners’* and scores of ’slightly better’, ‘better’ or ‘much better’ were reported for 73.20% of goals in this category. In addition, individual goals were prioritized and grouped with regard to personal importance. For 83.30% of the participants, the most important goal showed some improvement [*M* = 3.10, *SD* = 0.76 (*n* = 30)]. The mean score for all 109 goals at t_1_ was *M* = 3.00 (*SD* = 0.64), which is ’slightly better’. There was no statistically significant difference between scores at t_1_ and t_2_ (asymptotic Wilcoxon-Test: *z* = −0.843, *p* = .40). Good inter-coder reliability was reached for both the individual goals (93,2%) and the personal benefit (93,3%) as shown by comparing the analysis of the two coders.

**Table 5:** Categories and degree of change for individual hearing and communication goals measured by M-COSI at t1. Categories show the hearing and communication difficulties that AHL want to improve. (*N* = 30) (109 goals in total). .

Main categories	Subcategories	Number of goals	*M*	*SD*	*Range*
Adverse speech- and communication behavior of communication partners		41	3.07	0.85	2–5
	Unclear speech				
	Turned away speaking				
	Speaking at once/simultaneously				
	Speaking from spatial distance				
Difficult communication situations		37	2.81	0.85	2–5
	Conversation in group situations				
	Conversation in background noise				
	Conversation in adverse acoustic enviroment				
	Conversation in road traffic				
	Medical consultation/doctor-patient conversation				
Emotional burden		10	3.30	0.95	2–5
	Uncertainty concerning one's response behavior				
	Fear of poorer speech intelligibility				
	Feeling of isolation				
	Negative reactions of communication partners				
Speech intellegibility in social events		9	3.11	0.93	2–4
	Speech intellegibility in social events/theatre				
	Speech intellegibility in sport classes				
Telephone conversations		4	2.75	0.96	2–4
Listening to television		5	3.00	1.26	2–5
Listen to other signals		3	3.67	1.53	2–5

Degree of change for the goal categories with means (M), standard deviation (SD) and range. Results are based on a 5-point-scale with the answering options: worse (1), no difference (2), slightly better (3), better (4) and much better (5).Values >2 can be interpreted as improvement.

##### Overview of what participants liked about the ACE sessions and how they can be improved

The comments in Table 6 aggregate the feedback from all five sessions. Feedback results show that participants especially liked the ACE program content and the group approach. Nearly half of the comments indicate that no improvement was necessary. The main suggestions for improvement refer to including more interactive exercises, more time for exercises and group discussion, more information about hearing systems/technical matters, and implementing more psychosocial issues such as talking about one’s own hearing loss/hearing difficulties, self-disclosure of hearing loss, and feelings associated with hearing related communication problems.

**Table 6:** Overview of feedback sheets about the ACE sessions. .

Main category	Number of codings	Subcategory	Number of codings
**What did you like about the ACE sessions?** (144 comments and 273 codings in total)			
General positive comments	14		
Group atmosphere	47	Group atmosphere in general	15
		Communication behavior within the group	10
		Social interaction between participants	16
		Openess among participants	6
Methodical approach	69	Group approach/exchange in the group	24
		Course facilitator	23
		Integration of significant others	6
		Learning by use of example situations	6
		Shared work on course themes	5
		Visualisation and fixation of course content	3
		Home excersises	2
Dealing with the hearing loss	33	More awareness for the own hearing situation	12
		Self-efficacy in hearing situations	12
		Identifying needs and talking about them	4
		Dealing with hearing loss more openly	4
Course content	65	General course content	20
		To learn communication strategies	30
		Information about Assistive Listening Devices	11
		To learn about hearing loss in general and its impact	4
Course structure	28	Course structure	12
		Group composition	12
		Break as course element	4
**How could the ACE sessions be improved?** (106 comments and 115 codings in total)			
Nothing to improve	53		
Improvement of methods used	19	More interactive exercises	11
		Ensuring good communication behavior of participants	3
		Explaining exercises more clearly	3
		More visualisation of course content	2
Improvement of course organisation			
and structure	19	Improvement of room conditions	5
		More time for open exchange between participants	4
		Composition of participations	4
		More time in each session	3
		Better structure of module 5 (ALD)	3
Improvement of course materials	18	Providing of illustrative technical material in module 5 (ALD)	12
		Improvement of handouts	6
Improvement of course content	8	More psychosocial content	4
		Information about auditory processing	2
		More technical information about hearing systems	2

ACE, Active Communication Education; ALD, Assistive Listening Devices.

## Discussion

Overall, the results of this study show a positive impact of a German version of the ACE program in terms of a significant post-program improvement for communication strategy use (CSS of the CPHI) and for the acceptance of hearing loss (Accept total of the CPHI). In addition, the majority of goals identified by participants showed improvement (M-COSI), and overall positive outcomes were achieved as measured by the IOI-AI. Feedback on attending the program was also overwhelmingly positive, although areas for improvement were identified. One unexpected finding was the increased anxiety reported by participants on the Anxiety subscale of the HADS-D from pre- to post-program. No statistically significant changes were found on other outcome measures of hearing related emotional and social difficulties (HHIE/A), mental well-being (HADS-D), or health-related quality of life measures (SF-12).

These findings are here mainly discussed in comparison with other ACE evaluation studies of the original English version (Australia) (2), the Swedish version (5–7) and the Spanish version (Chile) (8). In those studies, sample sizes, participants’ages, degree of hearing loss, number of hearing-aid fitted persons, and gender distribution differed in various degrees from the current study. However, our discussion compares the findings of the ACE evaluation studies and the current studies, since these factors may underly the differences. Note that German CPHI number of items and response scales differed from the English and the Swedish CPHI versions, as described above (see Pre-Post Program Measures). The current results demonstrating an increased use of communication strategies are consistent with previous studies for the Australian and Swedish ACE versions (2, 5, 6).

Similarly, Habanec and Kelly-Campbell (4) found a statistically significant effect of improved communication strategy use in a group aural rehabilitation-training program. In their study, in spite of a smaller sample size (*N* = 24), a greater level of improvement was found. Study participants were about 10 years younger (*M* = 59.33), and hearing loss was smaller (*M* = 31.97 dB HL) than in the current study. The sample size, age of study participants, amount of hearing loss, and initial scores might be relevant influencing factors. These data all indicate that communication strategy use can be learned and enhanced by group training programs.

Although the Acceptance Scale of the CPHI has not been used in other studies of ACE outcomes, previous studies have reported qualitatively that acceptance of hearing loss is an outcome of the program. For example, Hickson, Worrall, and Scarinci (2) used an open-ended question about actions taken by participants after attending ACE. Many identified actions related to *Increased awareness of and acceptance of hearing* and being *More positive about hearing impairment*. In the ACE evaluation studies of Öberg (5) and Öberg, Bohn and Larsson (6), qualitative results also included *more awareness, self-confidence,* and *more open dealing with one's own hearing loss*. Acceptance of hearing loss has long been understood as an important precondition for positively coping with the condition and taking steps in the “patient journey” [see (34)].

The results of enhanced acceptance scores in this study underline the importance of the ACE program.

The M-COSI outcome showed a mean score of *M* = 3.0 (*SD* = 0.64), which only represents only a slight average improvement for the total of all hearing- and communication goals. The M-COSI total scores in the studies of the Swedish version were higher, and reached values of *M* = 3.41 (*SD* = 1.05) (6) or *M* = 3.49 (*SD* = 1.04) (5). In both evaluation studies for the Swedish version, younger persons were included, so age might be an influencing factor for higher scores in reaching hearing- and communication goals. [Ö berg, Bohn and Larsson (6): range of age = 39−82 years, *M* = 69.8, *SD* = 9.3; Öberg (5): (*N* = 77, range of age = 41−94 years aged, *M* = 73.9, *SD* = 9.8)]

Overall, the content of the M-COSI categories were similar to those of other ACE evaluation studies, and referred to communication skills, difficult communication situations, or knowledge about hearing loss (2, 5, 6). The categories of goals obtained with the M-COSI suggest that AHL need more than traditional device-focused rehabilitation to improve their everyday communication.

For many participants in the current study, identifying and developing individual goals on the M-COSI proved quite difficult, and they found it challenging to come up with goals that they could modify by changing their behavior. Jennings (35) previously pointed out the importance of individual goal-setting in behavior-based rehabilitation approaches, as they can increase motivation to change behavior and can be evaluated post-intervention. It was clear that participants in the current study became aware of their individual needs during the goal-setting process and, additionally, they had the opportunity to specifically evaluate individual issues. In future ACE courses and ACE program intervention studies, more support in the goal-setting process of the M-COSI should be implemented.

As measured by the HHIE/A, no statistically significant reduction of the perceived emotional or social hearing difficulties was found in this study. This is in contrast to results of previous evaluation studies of other ACE versions (5, 6, 8). It seems that participants in the current study came into the program with lower baseline scores of the HHIE/A than the participants in other studies, thus allowing less room for improvement. Several participants started the ACE program reporting few hearing-related difficulties, which implied floor effects. The age of study participants (5, 6), hearing-aid fitting [in (8) only non-hearing-aid users were included], and initial scores might also be relevant influencing factors.

However, after attending some ACE sessions, participants’ views often changed, and they became more aware of their hearing difficulties. Increasing awareness can be seen as a success for the communication training program, but might also lead to higher scores of the HHIE/A at t1/t2. To address this aspect, a methodological solution might be to differentiate between awareness of the person's own hearing difficulties on the one hand and the actually hearing difficulties on the other. Qualitative data of the personal benefit included a number of categories that were similar to HHIE/A items, such as *Reduction or prevention of communicative or social withdrawal*, and M-COSI categories like *Difficult communication situations* or *Speech intelligibility in social events*. These findings showed that participants perceived some improvement regarding their activity and participation.

A change in awareness of their own hearing difficulties may also explain the significant increase in anxiety found with the HADS-D immediately post-program. In contrast, the qualitative data showed that positive emotional and social effects were experienced by participants as the result of attending the ACE program (see Table 5). Results of ACE evaluation studies of the Swedish version from Öberg, Bohn and Larsson (6) and Öberg (5) differed from this. Significant improvement measured by the HADS was found for the depression scale and the total scale in Öberg, Bohn and Larsson (6). In comparison to the current study (see above), that study embraced a wider range of participants’ ages and included younger participants. In the multi-centre-study, a statistically significant improvement of HADS scores was found for study participants with moderate hearing loss (5).

Thus, crucial influencing factors for HADS Scores might be age and the degree of hearing loss.

Further research is necessary to understand the psychosocial changes occurring for participants who attend ACE. As suggested by Michaud & Duchesne (36), a further follow-up measurement after 9 months or later would be interesting to investigate the continuing development of the hearing difficulties and psychosocial outcomes.

The findings from the feedback sheets demonstrated that participants especially liked the group approach, and this is consistent with feedback on the original and the Swedish ACE programs (2, 5, 6). This is also consistent with the large body of evidence on the importance of group social interactions for older adults, and the positive outcomes it can have for health and well-being (37, 38).

The overall feedback indicated a high level of satisfaction with the content and methodological approach of the evaluated ACE program. Aspects that should be improved, such as more time for exercises, a higher number of interactive exercises, more technical information, or additional content that addresses psychosocial aspects of hearing loss were implemented in the final version of the German ACE program. Based on HADS-D findings with increased anxiety values and the feedback towards more psychosocial content, the final German ACE version includes a complete additional module that addressed psychosocial aspects of hearing loss, emotional reactions of hearing related communication difficulties, and the burden experienced by family members [see (39)].

The mixed-methods approach of collecting qualitative data (M-COSI categories and open-ended question for benefit) as well as quantitative data was a clear strength of the current study, as the qualitative data helped in clarifying various disclosing aspects, and helped in interpreting quantitative data.

## Limitations

Major limitations of this preliminary evaluation study revolve around the absence of (1) a control group, (2) the small sample size, and (3) the unequal distribution of gender. As intervention studies with the Swedish ACE showed that the effectiveness of the program differed significantly by gender (women experienced more benefit than men) (5), further research with a larger, more representative, sample size is needed to confirm the outcomes for the German version of the ACE. To compare communication program outcomes and treatment as usual, a waiting-list control group is recommended.

The intervention with data collection at Jade University took place between 2016 and 2017.

## Conclusions

The German version of the ACE program showed that participants’ communication strategy use and acceptance of hearing loss significantly improved post-program, and these positive outcomes were maintained 6 months later. Qualitative data underlined this outcome and indicated that participants had improved their abilities to cope with hearing and/or communication difficulties in everyday life, which is the aim of ACE (9). These first results for a German ACE version suggest that it could be a useful addition to clinical services for adults with hearing loss. The developed German ACE program as a standardized ICF- based communication training program for AHL and their family members fills a current gap in aural rehabilitation. The German ACE program can be provided in different settings like specialized clinics for aural rehabilitation, speech and language practices or other audiological/medical institutions. To investigate the clinical effects and benefits of ACE, further outcome measures to evaluate attitudes to aural rehabilitation, perceived self-efficacy, and social-communicative participation are recommended. For future studies, it would be interesting to analyze the influence of self-efficacy, attitudes to aural rehabilitation, hearing aid-users/non-users, duration of hearing aid use, degree of hearing loss, age, and the attendance of family members. The mixed-methods approach is strongly recommended to find out more about personal benefit and the patient journey of the AHL.

## Data Availability

The raw data supporting the conclusions of this article will be made available by the authors, without undue reservation.
